# A match made in mental space: Alignment between target skew and cue organization influences recall of attribute values

**DOI:** 10.3758/s13423-026-03018-y

**Published:** 2026-10-05

**Authors:** Patrik Sörqvist, Emil Skog, Alice Mason, John E. Marsh

**Affiliations:** 1 Department of Health, Learning and Technology, Luleå University of Technology, Laboratorievägen 14, 971 87 Luleå, Sweden; 2 Department of Building Engineering, Energy Systems, and Sustainability Science, University of Gävle, Gävle, Sweden; 3 Department of Psychology, University of Bath, Bath, UK; 4 Human Factors Laboratory, School of Psychology and Humanities, University of Lancashire, Preston, UK; 5 Faculty of Society and Design, Bond University, Gold Coast, QLD Australia

**Keywords:** Memory, Density, Distinctiveness, Comparison-induced distortion theory, Decision-making, Cue-based bias

## Abstract

Recall is often more successful for items from sparse (distinct) regions and less successful from dense (less distinct) regions in memory space. We propose that retrieval cues are also organized into crowded and less crowded regions depending on distinctiveness, and that successful retrieval depends on the distributional match between the organization of targets and cues. As a real-world test case, we used the labels from the European Union’s old (A+++, A++, A+, A, B, C, D) and new (A, B, C, D, E, F, G) energy-label scale as cues for memory retrieval of appliances’ kilowatt-hour (kWh) values. In a preregistered experiment, participants rated seven refrigerators, while studying associated kWh values drawn from a positive, a negative, or a non-skewed distribution. Ratings confirmed that the labels of the old scale were psychologically skewed in mental space whereas the new scale had a more linear organization. After a brief filled delay, labels served as retrieval cues for kWh recall. Analyses of recall patterns, complemented by analyses of recall error, showed that the linear cue scale yielded recalled values that were closer to the correct kWh values when target values were non-skewed, but recalled values were overly linear when targets were skewed. Conversely, recall was most successful with the skewed cue scale when target values were positively skewed. These findings suggest that retrieval cues constrain reconstruction by imposing a distributional structure on target representations, and that recall is most successful when this structure aligns with the organization of encoded values in mental space.

**Supplementary Information:**

The online version contains supplementary material available at https://doi.org/10.3758/s13423-026-03018-y.

## Introduction

In cued recall, a cue is associated with a target at encoding, and participants must retrieve the correct target when later presented with a given cue. Successful recall depends on, for example, trace-cue compatibility (Nairne, 2002), set size and stimulus context (Haridi et al., 2026), contextual interference (Park et al., 2006), and the diagnostic value of cues (Goh & Lu, 2012). In accordance with distinctiveness theories of memory (Brown et al., 2009; Hunt, 1995, 2006; Hunt & Lamb, 2001; Hunt & McDaniel, 1993; Kowialiewski et al., 2024; Neath et al., 2006), the uniqueness (Mäntylä & Nilsson, 1988) and distinctiveness (Brandimonte & Passolunghi, 1994) of retrieval cues determine successful recall, with more distinct cues leading to better recall, especially when the same distinct cue (e.g., an unfamiliar odor) is presented both at encoding and at retrieval (Herz, 1997). Similarly, the distinctiveness in the memoranda determines successful recall, with distinct target items being easier to recall (Hunt & Smith, 1996; Neath et al., 2006; Schmidt, 1985; Skog et al., 2026; Smith & Hunt, 2000; Waddill & McDaniel, 1998). This raises the question of how distinctiveness of cues and distinctiveness of targets interact. Memory models such as Decision by Sampling (Brown & Mathews, 2011; Stewart et al., 2006), SIMPLE (Brown et al., 2007), and REM (Shiffrin & Steyvers, 1997) illustrate that items in crowded regions of psychological space (locations where target items are less distinctly stored) are harder to discriminate and recall, compared to items from less crowded regions. We propose that retrieval cues are also organized into crowded and less crowded regions depending on their distinctiveness. Critically, we also propose that memory retrieval depends on the match between how targets and cues are organized in mental space.

One way to study effects of *target*-distinctiveness on recall is to compare recall of items drawn from a positively skewed versus a negatively skewed stimulus distribution (Choplin & Hummel, 2002; Stewart et al., 2006; Wedell, 1996; Wedell et al., 2020). For instance, Choplin and Wedell (2014) asked participants to study a list of hamburgers and their associated calories. The calorie distribution was either positively skewed (i.e., many small and few large values) or negatively skewed (i.e., many large and few small values), with some values being identical across the two conditions. When later cued by hamburger labels, several systematic biases emerged. For example, an intermediate value (743.5 calories), present in both distributions, was overestimated in the positive skew condition and underestimated in the negative skew condition. Thus, memory shifted away from the distributional mean – resulting in a contrast effect. Similar distribution-density effects are found in stimulus judgment (Birnbaum & Rouvere, 2023; Huttenlocher et al., 2000; Parducci, 1965; Ungemach et al., 2011), with judgments being generally higher for negatively skewed stimulus distributions in comparison with positively skewed stimulus distributions, even when the two stimulus distributions’ sums and means are identical (Skog & Sörqvist, 2025; Skog et al., 2025).

**Fig. 1:**
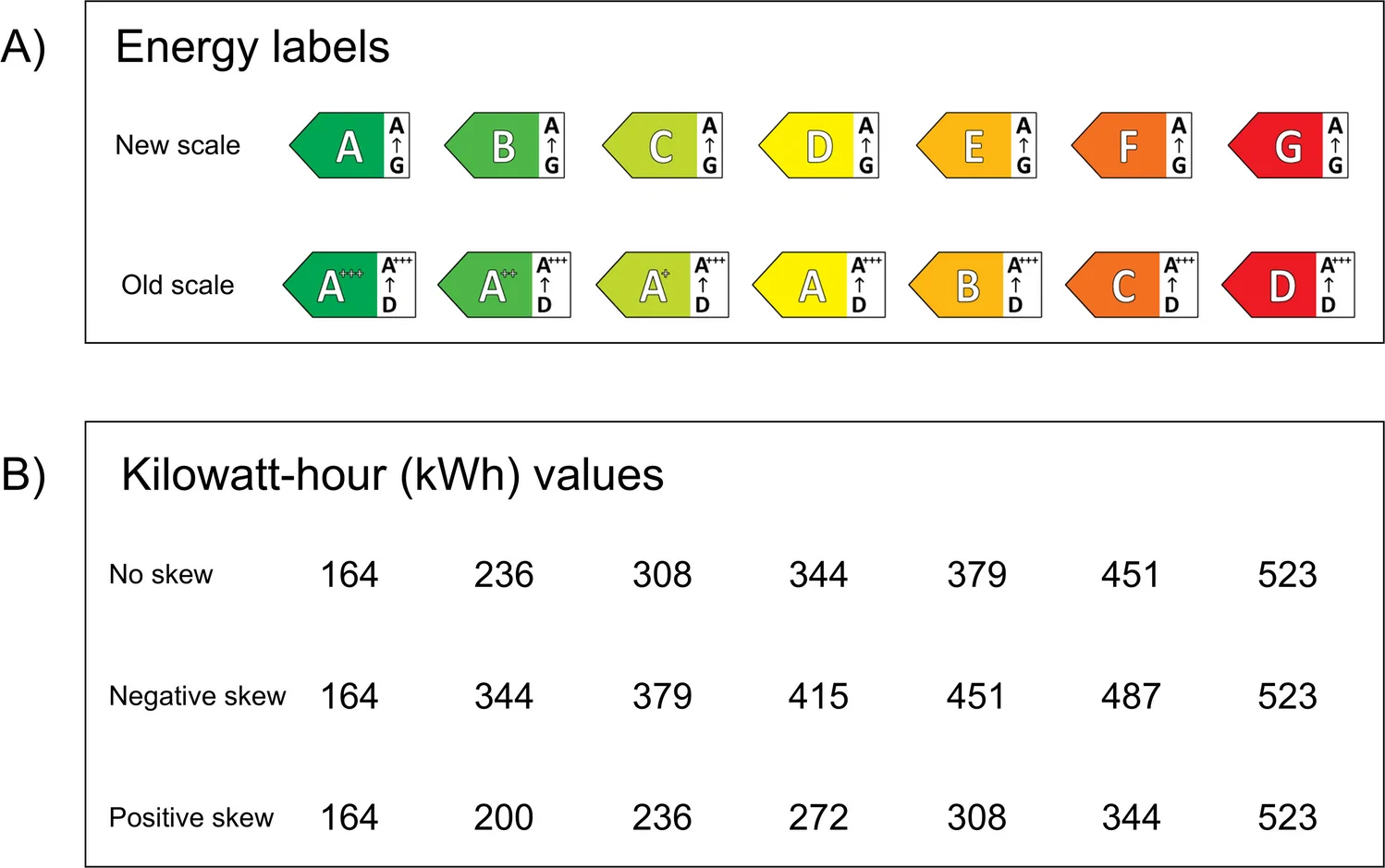
Stimulus material. The material is split by two experimental factors: Scale of energy labels (panel **A**) and Skewness of kilowatt-hour values (panel **B**)

We propose that retrieval cues can similarly be organized into positively or negatively skewed distributions in mental space, wherein *cue*-distinctiveness is higher in some regions and lower in other regions of the same cue set. To illustrate, consider the European Union’s (EU’s) scale of energy classes which is used to classify appliances based on their energy demand. Until recently, a scale with seven energy classes (A+++, A++, A+, A, B, C, and D) was used (Fig. 1), where A+++ is assigned to appliances with small energy demand (a low kilowatt-hour (kWh) value) and D to appliances that use the most kWh. The EU revised the energy label scale in 2021 and eliminated the ‘+’ categories. In this new scale, the seven energy classes are A, B, C, D, E, F, and G. An important feature of the old scale is that the most energy efficient classes (‘A+++’, ‘A++’, ‘A+’, and ’A’) are all perceived as relatively similar and high in environmental friendliness, whereas the other classes (‘B’, ‘C’, ‘D’) on the same scale are perceived as more distinct from each other (Skog & Sörqvist, 2026; see also Heinzle & Wüstenhagen, 2012; Stasiuk & Maison, 2022). Because of this, this old scale of energy classes can be characterized as skewed in mental space, in such a way that it is denser in the energy-efficient region and less dense in the inefficient region. In turn, the new scale (with energy classes ‘A’, ‘B’, ‘C’, ‘D’, ‘E’, ‘F’, ‘G’) is linearly ordered (or non-skewed) in mental space. We refer to the skewness of labels (retrieval cues) as its *structure* in mental space. This structure can be linear (as with the EU’s new scale) or skewed (as with the EU’s old scale). Distinctiveness is seen as a local property of this global structure. In skewed structures, parts of the cue set are dense (cues are here less distinct) and parts are sparse (cues are here more distinct). In linear structures, the cues are evenly spaced without less distinct parts.

**Fig. 2:**
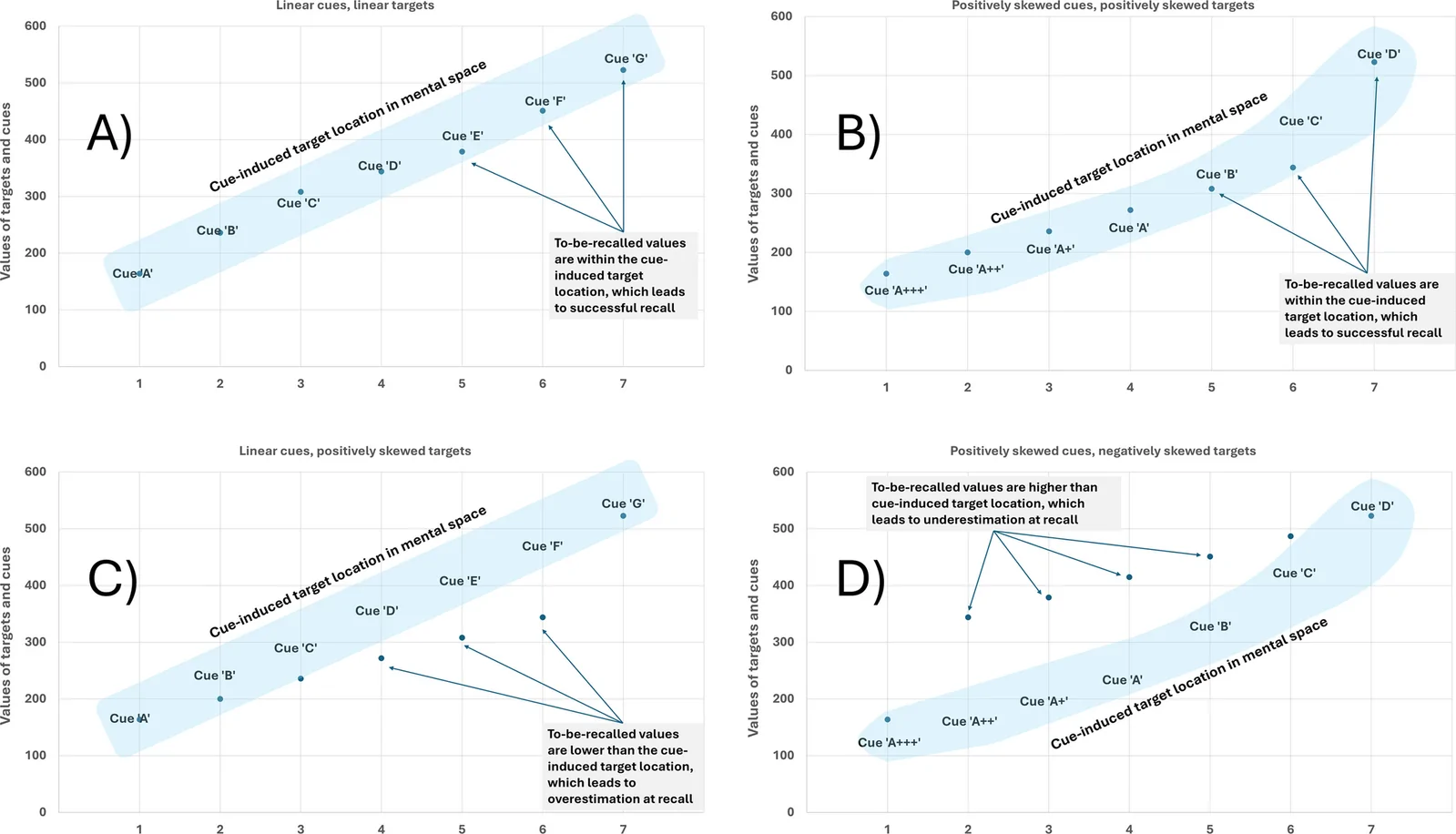
Summary of the predictions of a cue-target alignment model. Recall is successful when to-be-recalled values (targets) are organized within the cue-induced target location in mental space (area shaded blue), as when cues and targets are linearly spaced (**panel A**) and when cues and targets are both positively skewed (panel **B**). When cues are linear, but targets are positively skewed, the cue-structure produces overestimation (panel **C**). When cues are positively skewed, but targets are negatively skewed, the cue-structure produces underestimation (panel **D**)

According to the view that both *cues* and *targets* can be distributed linearly or skewed, their distribution-skew can either match (e.g., both are linear) or mismatch (e.g., cues are skewed, but targets are linear). What happens when the structure of retrieval cues and the skew of to-be-recalled values mismatch in mental space? Existing work on distribution-density effects (e.g., Choplin & Wedell, 2014; Wedell et al., 2020) has primarily treated retrieval cues as an ordinal scaffold against which target values are reconstructed. Here we extend this logic by testing whether the cue scaffold itself – specifically, the psychological spacing of label categories – influences recall. And we test whether recall depends on the alignment between (1) the skewness implied by the cue distribution in mental space and (2) the skewness of the to-be-recalled values. Our predictions are outlined in Fig. 2.

The comparison-induced distortion theory (Choplin & Hummel, 2002, 2005; Choplin & Wedell, 2014) provides a framework for understanding how cue structures influence memory reconstruction. According to this account, individuals interpret retrieval cues semantically (e.g., ‘cheeseburger’ vs. ‘bacon cheeseburger’) and generate relative comparisons between them (e.g., inferring that a cheeseburger has fewer calories than a bacon cheeseburger). These comparisons imply expected differences between items – referred to as comparison-suggested differences (D) – which are then used to guide reconstruction of uncertain values.

A key implication of the comparison-induced distortion theory is the semantic incongruity hypothesis (Choplin & Hummel, 2002), which proposes that recall accuracy depends on the correspondence between D and the true differences among target values. When comparison-suggested differences align with actual value differences, reconstruction is relatively accurate. However, when they diverge, recall becomes systematically biased. In such cases, we hypothesize that the memory system relies on the implied structure of the cues, leading estimates to shift toward that structure.

This distortion is particularly evident when target values are drawn from skewed distributions. Because the spacing implied by verbal comparisons may not match the true distribution, densely clustered values tend to be displaced toward sparser regions (e.g., lower values are overestimated in positively skewed distributions, while higher values are underestimated in negatively skewed distributions). Thus, mismatches between cue spacing and target spacing produce predictable reconstruction biases.

We tested a novel prediction based on this logic: *The skewness of the retrieval cue distribution* should influence recall. This leads to several hypotheses (Fig. 2): when people recall kWh values associated with energy labels, and the energy labels act as retrieval cues, the structure of retrieval cues in mental space (linear vs. skewed) will effectively organize target-value reconstruction. Most importantly, there should be an interaction between the skew of retrieval cues (i.e., the energy labels) and the skewness of the target items. One possibility is that systematic bias in recall depends on the alignment of the retrieval cues and the to-be-recalled items in mental space (Fig. 2), because when the target-skew and the cue-skew mismatch, the cues will hinder target reconstruction, but when target-skew and cue-skew match, the cues will facilitate reconstruction. The reason is that the comparison-suggested difference (D) between cues (e.g., ‘A+’ and ‘A’) matches the true difference in target values (e.g., a small difference in kWh attribute values) when the skew of the retrieval cues and the skew of the target values match, but D mismatches the true difference when cue-skew and target-skew mismatch (e.g., the psychological distance between ‘A+’ and ‘A’ suggests a small difference in kWh values, but the target-skew implies a larger difference). We tested these predictions in an experiment where participants studied kWh values with different skews (positive, negative, or non-skewed) along with their associated energy classes (taken from either the skewed/old or linearly ordered/new energy scale) and later recalled the kWh values after a brief delay.

## Method

### Participants

Participants were recruited via Prolific (www.prolific.com). An a priori power analysis in G*Power (Faul et al., 2007) was used to estimate the required sample size. Our goal was to obtain .80 power (two-tailed) to detect a medium effect size of Cohen’s *d* = 0.536 at the standard .05 alpha error probability.

Our estimate of effect size was based on a pilot study that focused on differences between the old and new energy label scales in a separate memory task examining recall biases for energy-labelled appliances. This pilot study revealed differences in recall bias in the upper parts of the scales corresponding to an effect size of Cohen’s *d* = 0.536. Although pilot-based effect-size estimates can be unstable, we used this estimate as a pragmatic guide for determining a sufficiently sensitive sample size. Based on this estimate, the a priori power analysis suggested a minimum sample of 56 participants in each scale condition (old vs. new scale). Because the present design additionally included three skewness conditions (2 × 3 = 6 groups), we aimed to recruit 56 participants in each of the six groups, resulting in a substantially larger total sample than is typically used in related paradigms.

A total *N* = 359 (178 female, 181 male; mean age = 46.7 years, *SD* = 13.2) were recruited but 23 participants (6%) were excluded from analysis as they fulfilled at least one criterion for exclusion (see Procedure below). The excluded participants were replaced to fulfill our pre-registered stopping rule of supporting our analysis with *N* = 56 in each of the six groups. The final analyzed sample was therefore 336 participants. All participants were based in the UK and had English as their first language. They reported having normal or corrected-to-normal vision and no color-blindness. All participants (including those whose data were excluded from analysis) were compensated at a per-hour rate of £9 for a total study time of approximately 7–8 min.

### Materials

The experiment was created in PsychoPy (version 2024.2.2; Peirce et al., 2019) and JavaScript and was made available online on Pavlovia. Participants used their own desktop computers, in an uncontrolled environment, to access the web-based experiment via Prolific. Participants used their own mouse and keyboard for responses.

The stimulus material (Fig. 1) consisted of 14 energy labels (seven in each of two scale conditions) and 21 kWh values (seven in each of three skewness conditions, inspired by Choplin & Wedell, 2014). Energy labels were of the “Class arrows” type, made available by the EU in PNG format on the file exchange tool CIRCABC. In the experiment, an energy label and a kWh value were paired with a PNG image of a refrigerator that was created with the assistance of a web-based artificial intelligence service. The refrigerator image was always the same, and thus not informative for the tasks. The refrigerator was presented in the middle of the screen, scaled to 40% of the participant’s monitor’s height (1 : 1 aspect ratio). Energy labels were shown to the immediate upper right of the refrigerator image, with a height corresponding to 10% of the participant’s monitor (5:3 aspect ratio). kWh values were presented below the energy labels and were followed by the suffix ‘kWh’ presented below the number. On each trial, one energy label and one kWh value were presented with the refrigerator image.

### Design

The study was a mixed 2 (Scale: new, old) × 3 (Skewness in kWh values: no skew, negative skew, positive skew) × 7 (Energy class: 1 st, 2nd, 3rd, 4th, 5th, 6th, 7th) design. Scale was a between-participants factor, where half of all participants were shown the new scale (A–G), and the other half were shown the old scale (A+++ to D). Skewness in the distribution of kWh values was also a between-participants factor where participants encountered different kWh values depending on the condition. See Fig. 1A for the energy labels in each scale condition and Fig. 1B for the kWh values in each skewness condition. In the ‘no skew’ condition, participants encountered kWh values that were not skewed, with a middle value of 344 kWh. Here, there were equally many values above as below the middle (344). In the ‘negative skew’ condition, participants encountered a distribution of kWh values that was negatively skewed, i.e., with more values above the middle (344). The ‘positive skew’ condition contained the opposite pattern of stimulus values, with more values below the middle (344). As Fig. 1B shows, all three skewness conditions contained the same lowest (164) and highest values (523), and all distributions contained the value 344. Energy class was a within-participants factor, where all participants completed trials with all seven energy classes in a given scale condition (Fig. 1A) and with all seven kWh values in a given skewness condition (Fig. 1B). The pairing of energy labels and kWh values was always matched according to the horizontal layout of Fig. 1 (e.g., the first energy label was always paired with the first kWh value, and so on).

### Procedure

The experiment was advertised on Prolific as a task of evaluating then recalling the energy requirements of different refrigerators. Participants were told that they would complete a rating task followed by a memory test, and that further instructions would follow within the experiment. Participants provided informed consent upon starting the experiment. There were two instruction screens at the beginning of the experiment. The first screen stated that all the refrigerators will look the same, but that some are more energy efficient than others. This also explained that a refrigerator is given an energy label that represents its energy demand. A graphical representation of the full scale of seven energy classes was shown, making the seven steps of the scale explicit and revealing the color coding of the labels. Here, text stated that the most efficient energy rating is ‘A’ (or ‘A+++’, depending on condition), shown in green, and that the least efficient energy rating is ‘G’ (or ‘D’, depending on condition), shown in red. The second instruction screen introduced information about kWh values as an indicator of a refrigerator’s annual energy demand. Participants were told that each refrigerator will have both an energy label and information about its kWh annual energy consumption, and that kWh would be represented by a number. Participants were also made aware that there would be multiple parts to the experiment, and that the final part was a task to recall from memory the kWh numbers of each refrigerator.

Participants then took part in three experimental phases:

1. An encoding phase,
2. A filled retention interval, and
3. A recall phase.

In the encoding phase, participants completed rating tasks where they engaged with each refrigerator and their associated energy label and kWh value. They rated each of the seven refrigerators (which were characterized by their energy label and kWh value) in three separate rating tasks, affording three encoding events per stimulus. The order of presentation for the seven stimuli was randomized anew in each task and uniquely for each participant. The three rating tasks asked participants to rate (1) environmental friendliness, (2) running cost, and (3) willingness to pay. The rating tasks were completed sequentially in this order by all participants. There were paused instruction screens before each new rating task. Participants responded by clicking an 11-point slider scale presented at the bottom of the screen. In all three rating tasks, the left- and rightmost slider response options were associated with a ‘worst’ and ‘best’ response, respectively (rating task 1: ‘Not environmentally friendly’ and ‘Very environmentally friendly; rating task 2: ‘Much higher cost than average’ and ‘Much lower cost than average’; rating task 3: ‘I would be willing to pay very little’ and ‘I would be willing to pay very much’).

Upon completing the rating tasks of the encoding phase, participants were instructed to answer a few math questions with a limited time for responses. This was a filled retention interval task, situated between the encoding and recall phases. Six sequential math questions were presented for a fixed duration of 10 s each. Questions were in the form: ‘Is (6 × 3) + 2 = 16?’. Participants answered yes or no by button press as a progress bar indicated the time remaining for a response. Responses to this filler task were not of theoretical interest, but participants responded on 100% of trials (zero missing responses across all 2,016 trials) with a mean accuracy of 94.9%, indicating broad engagement with the task.

Upon finishing the 60-s filled retention interval task, participants were then told that the final part of the experiment was next. Their task was now to recall from memory the kWh values of each refrigerator. The refrigerators and their associated energy labels were presented in sequence across seven trials, in a random order. The information about kWh values was hidden from participants. On each memory trial, participants were prompted to type a number to respond what the kWh value of a displayed refrigerator was. Participants typed this number in a text box below the energy label, in the screen location where the kWh value was previously presented during the encoding phase. The text box allowed free text typing, but participants were asked to only type using number characters, and to submit their answer with the enter (return) key.

Upon completing the memory test, participants were asked yes/no control questions, while being assured that their answers to these questions would not impact their monetary compensation. Later, these control questions were used to identify that seven participants had either restarted the experiment or had cheated on the memory test by taking notes of the refrigerators during the encoding phase. These seven participants were replaced and their data not analyzed. An additional 16 other participants were replaced due to them failing to type kWh numbers as responses in the memory test, or they typed answers which indicated that they had not understood the stimuli or the task. One such exclusion criterion was to exclude participants who had not typed any three-digit numbers. We considered this an indicator of misunderstanding as all kWh values were three-digit numbers. In total, we excluded 23 participants from analysis who were replaced to satisfy our pre-registered stopping rule for data collection (we aimed to support our analysis with *N* = 56 in each group). At the final stage of the experiment, participants were shown a debrief outlining the purpose of the experiment, before being taken back to Prolific.

## Results

**Fig. 3:**
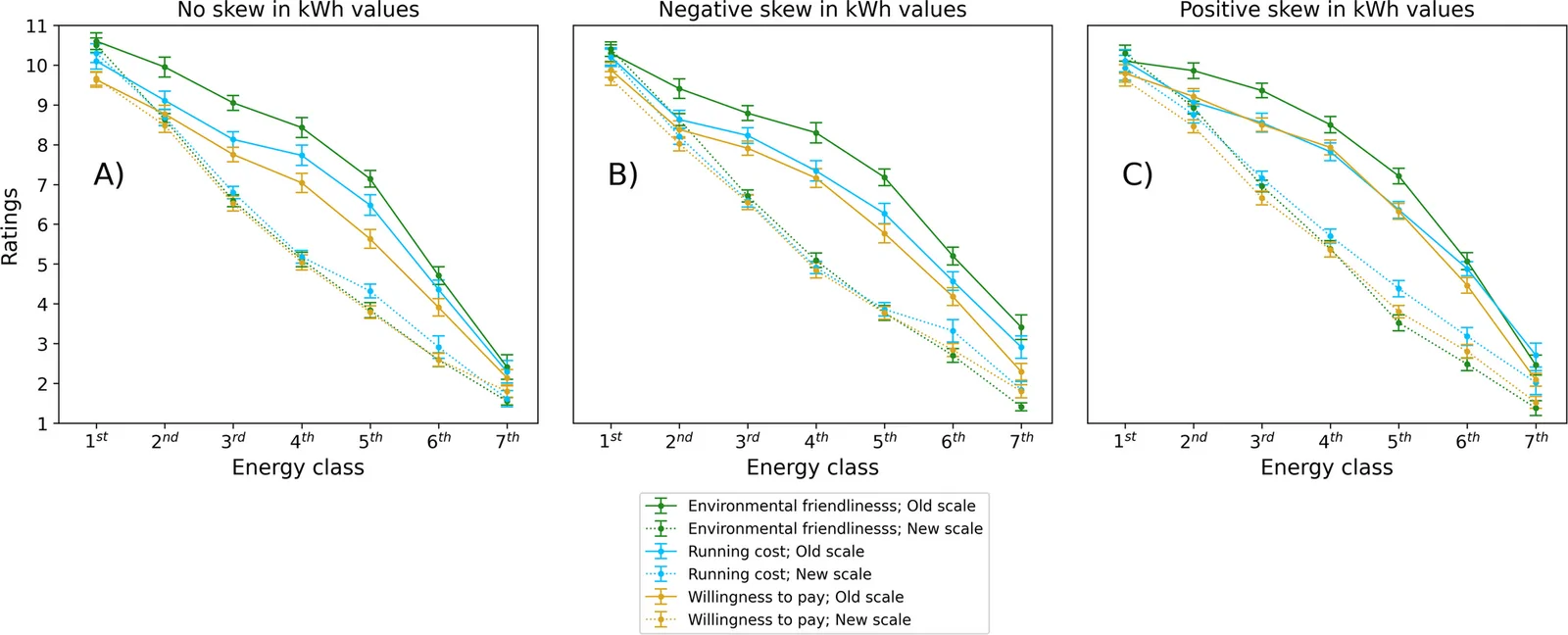
Ratings from the encoding phase of the experiment (three rating tasks). Error bars represent the standard errors of the means

Three rating tasks constituted the encoding phase of the experiment. The first rating task (environmental friendliness) was of primary interest, as this directly taps the environmental dimension of judgment. It was analyzed with a repeated-measures 2 (Scale: new, old) × 3 (Skewness in kWh values: no skew, negative skew, positive skew) × 7 (Energy class: 1 st, 2nd, 3rd, 4th, 5th, 6th, 7th) analysis of variance (ANOVA) that was adjusted with a Greenhouse-Geisser correction. A main effect of scale, *F*(1, 330) = 464.5, *p* <.001, ${\eta}_{p}^{2}=.585$, and an interaction between energy class × scale, *F*(3.39, 1119.31) = 81.23, *p* <.001, ${\eta}_{p}^{2}=.198$, revealed differences across the scales. As seen in Fig. 3, the A+ classes in the old scale were all seen as quite environmentally friendly, whereas the other energy classes were more distinguished in terms of their environmental valence. There was no three-way interaction, *F*(6.78, 1119.31) = 1.74, *p* =.099, ${\eta}_{p}^{2}=.010$, showing that skewness in kWh values did not significantly alter the effects of scale across energy classes, suggesting that scale was a more influential stimulus feature than skewness (Fig. 3). For completeness, we also report that there was a main effect of energy class, *F*(3.39, 1119.31) = 1774.61, *p* <.001, ${\eta}_{p}^{2}=.843$, no main effect of skew (*F* < 1), and an interaction between energy class × skew, *F*(6.78, 1119.31) = 2.56, *p* =.014, ${\eta}_{p}^{2}=.015$, but no interaction between scale × skew (*F* < 1).

**Fig. 4:**
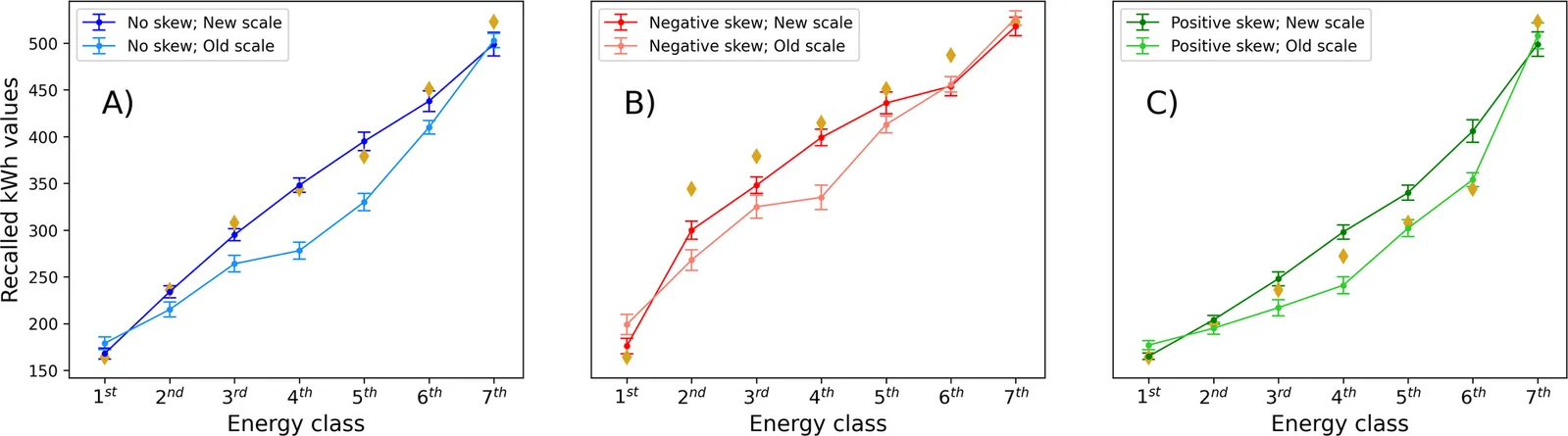
Recalled kilowatt-hour (kWh) values. Panels show different skewness conditions. Gold diamonds show the true kWh values. Error bars represent the standard errors of the means

The recall phase of the experiment tasked participants to recall the kWh values, using energy labels as retrieval cues. We begin by analyzing the effects of scale. Recalled kWh values are shown in Fig. 4, and were analyzed with a repeated-measures 2 (Scale: new, old) × 3 (Skewness in kWh values: no skew, negative skew, positive skew) × 7 (Energy class: 1 st, 2nd, 3rd, 4th, 5th, 6th, 7th) ANOVA that was adjusted with a Huynh-Feldt correction. A main effect of scale, *F*(1, 330) = 18.41, *p* <.001, ${\eta}_{p}^{2}=.053$, energy class, *F*(5.09, 1680.51) = 1540.25, *p* <.001, ${\eta}_{p}^{2}=.824$, and an interaction between energy class × scale, *F*(5.09, 1680.51) = 22.52, *p* <.001, ${\eta}_{p}^{2}=.064$, revealed differences across the scales that were like the effects on ratings of environmental friendliness. As seen in Fig. 4, recalled kWh values in the upper part of the old scale (A+++, A++, A+, A) showed greater compression than those in the corresponding region of the new scale (A, B, C, D). This, taken together with the findings from the encoding (rating) task, indicates indistinctiveness or compression in mental space.

For the analysis of the effects of scale, we consider the ‘no skew’ condition in particular (Fig. 4A), as this was not biased by skewness of the to-be-recalled items. As Fig. 4A shows, the new scale produced recall values that were closer to the true values than the old scale, and the old scale was biased towards underestimations. These underestimations maximized in the middle of the scale (recall of the middle value, 344), where the new scale group’s mean answer was 348 and the old scale group’s mean answer was 278 (Fig. 4A). When the to-be-recalled values were negatively skewed (Fig. 4B), the new scale produced recall values that were closer to the true values than the old scale did, but the recalled values were still overly linear when cued by the new scale. In turn, when the to-be-recalled values were positively skewed (Fig. 4C), the old scale produced recall values that were closer to the true values, while the new scale again produced overly linear recall.

The to-be-recalled kWh values varied across the three skewness conditions (Fig. 1B). Skewness thus produced a main effect, *F*(2, 330) = 62.33, *p* <.001, ${\eta}_{p}^{2}=.274$, and an interaction between energy class × skewness, *F*(10.18, 1680.51) = 15.39, *p* <.001, ${\eta}_{p}^{2}=.085$, but no interaction between scale × skewness (*F* < 1). Importantly, there was a smaller but significant three-way interaction, *F*(10.18, 1680.51) = 1.91, *p* =.038, ${\eta}_{p}^{2}=.011$, indicating that the energy labels – which were used as retrieval cues – had slightly different effects depending on the skewness of the kWh values.

Because the to-be-recalled kWh values differed across skewness conditions by design, analyses of raw recalled values necessarily reflect both differences in the stimulus sets and systematic reconstruction in memory. To isolate recall bias (signed error) and precision (absolute error) independently of the stimulus distributions, we conducted complementary analyses of signed error (response minus the correct kWh value) and absolute error (the absolute deviation from the correct kWh value). These analyses are reported in full in the Online Supplementary Materials and confirm that the effects described above reflect systematic bias patterns rather than artefacts of the underlying value distributions.

## Discussion

We tested how the match between *cue*-distribution and *target*-distribution influences recall. Ratings at encoding confirmed that retrieval cues (energy labels) of the old EU scale are compressed/indistinct at the highest-efficiency categories (‘A+++’, ‘A++’, ‘A+’ ‘A’), indicating non-uniform cue spacing in psychological space. In turn, retrieval cues of the new EU scale (‘A’, ‘B’, ‘C’, ‘D’, ‘E’, ‘F’, ‘G’) are evenly spaced. When these labels were used as cues for retrieval of kWh target values with various skew, recall was best when the skew of the cues (linear versus skewed) and the skew of the targets (linear, positively skewed, negatively skewed) matched. The results revealed that when cues implied evenly spaced steps between targets (energy labels A–G), recall showed reconstruction toward equal intervals, which led to successful recall of non-skewed target values (see Online Supplementary Materials for corresponding error analyses) but produced bias when target values were skewed. With skewed target values and linear cues, recall became overly linear, because reconstruction becomes regularised toward evenly spaced cue intervals. Conversely, when cues implied compressed spacing at the top end (energy labels ‘A+++’ to ‘A’), recall was more successful for positively skewed target values, but impaired recall of non-skewed or negatively skewed targets. Collectively, the results support an alignment account in which cue structure constrains target reconstruction. Importantly, this effect is not tautological, because cue structure and target structure were manipulated independently. Alignment therefore reflects a testable relation between two distinct representational systems – on the one hand the psychological organisation of retrieval cues and on the other hand, the distributional organisation of encoded target values.

Previous research has extensively studied distribution-density effects of target values on judgment (Birnbaum & Rouvere, 2023; Huttenlocher et al., 2000; Parducci, 1965; Skog & Sörqvist, 2025; Skog et al., 2025; Ungemach et al., 2011) and recall (Choplin & Hummel, 2002; Choplin & Wedell, 2014; Stewart et al., 2006; Wedell, 1996; Wedell et al., 2020). Our results extend previous research by showing that distribution-density effects are modulated by retrieval cue distribution. When skewed to-be-recalled values are encoded with linear cues (as implied by the EU’s new ecodesign scale), this cue structure creates an overly linear distribution of reconstructed values at test. Similarly, when the cue structure is positively skewed (as implied by the EU’s old ecodesign scale) and is paired at encoding with negatively skewed to-be-recalled values, this cue structure results in a comparably large bias at test. Conversely, when both cue-structure and target-structure are positively skewed, this cue-structure facilitates value reconstruction at test.

Our proposed alignment account suggests that retrieval cues can constrain reconstruction by imposing a relational structure on target representations. In this sense, retrieval cues do not merely provide access to stored values but actively influence the geometry within which reconstruction occurs. Within this framework, distinctiveness is a local property of that structure. Organization and distinctiveness are closely linked: items are easier to recall when they are both organized and distinct within mental space (Hunt & Smith, 1996). Similarity-based comparisons provide a mechanism for this effect, as individuals reconstruct item values by evaluating their relationships with neighbouring items in memory (Choplin & Hummel, 2002; Medin et al., 1993). Reinstating an appropriate organizational structure at retrieval allows distinctiveness to emerge at the level of individual items, producing more precise recall (Smith & Hunt, 2000). Importantly, our results show that low distinctiveness is not inherently detrimental. The compressed ‘A’ classes of the old scale produced more successful recall when the skew of the kWh values matched the skew of the labels (positive skew), but impaired recall when cue-skew and target-skew misaligned. This demonstrates that the effect of low distinctiveness depends on whether the organization of retrieval cues aligns with the organization of the to-be-recalled items.

These findings also connect to broader theoretical frameworks. Decision by Sampling (DbS; Brown & Mathews, 2011; Stewart et al., 2006) predicts that recalled or estimated values depend on both the rank and the range of items in memory, and when relative discriminability is considered, the model can account for distribution-dependent biases. Similarly, memory models such as SIMPLE (Brown et al., 2007) and REM (Shiffrin & Steyvers, 1997) illustrate that items in crowded regions of psychological space are harder to discriminate and recall, producing compression effects similar to those we observed. However, neither DbS nor comparison-induced distortion theory (Choplin & Hummel, 2002; Choplin & Wedell, 2014) explicitly addresses how the *cue*-distribution interacts with the *target*-distribution to shape memory and evaluation. Together, these perspectives suggest that our results reflect the combined influence of cue organization and target discriminability, highlighting how the mental distribution of cues and targets shapes memory and bias.

In line with the comparison-induced distortion theory, we have interpreted our findings primarily in terms of retrieval processes. However, the present design does not allow a clear separation between encoding- and retrieval-based mechanisms. The energy labels were present during encoding as well as at recall, making it possible that the structure of the retrieval cues also shaped how the kWh values were initially encoded and organized in memory. Ordinal labels may impose an organizational framework already at encoding, influencing how items are compared, compressed, or differentiated in mental space. Consequently, the observed alignment effects may reflect a combination of encoding and retrieval processes rather than retrieval alone. Future research could disentangle these mechanisms by manipulating the availability or structure of labels at encoding and retrieval independently. More broadly, the present findings suggest that retrieval cues do not merely provide ordinal access to stored values but actively constrain reconstruction by imposing a relational structure on the target space.

We end with a brief note on policy implications, given the real-world stimuli used in the present study. Choice alternatives often carry labels that are intended to help people perceive, interpret, compare, and remember attribute values (e.g., calories and vitamins; Jiang et al., 2025; carbon footprint; Thøgersen & Nielsen, 2016; kWh values of appliances; Waechter et al., 2016). Labels are often arranged on a graded scale, which can be linearly ordered (e.g., A–G) or non-linearly ordered (e.g., supersize vs. standard vs. ultra-thin). Linearly ordered categories may imply that the underlying values are also linearly spaced, but this is not always the case. For instance, the difference in carbon footprint between a green and yellow eco-label may not equal the difference between a yellow and red label (Fresacher & Johnson, 2023). We show that eco-label design should take the relation between label categories and underlying values into consideration. Otherwise, people may systematically misremember underlying values in ways that exaggerate or understate differences between options.

## Supplementary Information

Below is the link to the electronic supplementary material.

Supplementary file1 (PNG 191 kb)

High resolution image (TIFF 665 kb)

Supplementary file2 (PNG 180 kb)

High resolution image (TIFF 671 kb)

Supplementary file3 (PNG 155 kb)

High resolution image (TIFF 391 kb)

Supplementary file4 (DOCX 574 KB)

## Data Availability

We declare our hypotheses, methods, analyses (statistical tests conducted in Jamovi, version 2.3.28.0), and results. The experiment was pre-registered. The data and pre-registration supporting this article are publicly available via the Open Science Framework (https://doi.org/10.17605/OSF.IO/H3YTN).
