# Supplementary material for: A match made in mental space: Alignment between target skew and cue organization influences recall of attribute values
Source: Psychon Bull Rev. 2026 Oct 5;33(8):249. doi: 10.3758/s13423-026-03018-y (PMC13638717; doi:10.3758/s13423-026-03018-y)
Supplement: Supplementary file 1 [file 13423_2026_3018_MOESM4_ESM.docx]

**Supplementary Materials**

*Figure S1*. Bias in recalled kWh values (responses – correct answers). Gold line marks 0 on the y-axis: the correct answers.

*Table S1*: Results of a repeated measures ANOVA on bias in recalled kWh values (responses – correct answers).

| Factor | *df* | *F* value | *p* value | η^2^_p_ |
| --- | --- | --- | --- | --- |
| Energy class | 5.09, 1680.51 | 23.98 | < .001 | .068 |
| Scale | 1, 330 | 18.41 | < .001 | .053 |
| Skew | 2, 330 | 12.18 | < .001 | .069 |
| Energy class × Scale | 5.09, 1680.51 | 22.52 | < .001 | .064 |
| Energy class × Skew | 10.18, 1680.51 | 15.03 | < .001 | .084 |
| Scale × Skew | 2, 330 | 0.53 | .587 | .003 |
| Energy class × Scale × Skew | 10.18, 1680.51 | 1.91 | .038 | .011 |

*Figure S2*. Absolute deviations from correct answers (absolute values of responses – correct answers).

*Table S2*: Results of a repeated measures ANOVA on absolute deviations from correct answers.

| Factor | *df* | *F* value | *p* value | η^2^_p_ |
| --- | --- | --- | --- | --- |
| Energy class | 5.43, 1790.47 | 14.51 | < .001 | .042 |
| Scale | 1, 330 | 4.60 | .033 | .014 |
| Skew | 2, 330 | 2.88 | .057 | .017 |
| Energy class × Scale | 5.43, 1790.47 | 14.81 | < .001 | .043 |
| Energy class × Skew | 10.85, 1790.47 | 7.52 | < .001 | .044 |
| Scale × Skew | 2, 330 | 3.03 | .050 | .018 |
| Energy class × Scale × Skew | 10.85, 1790.47 | 1.77 | .055 | .011 |

*Figure S3:* Recalled kilowatt-hour (kWh) values from the ‘no skew’ condition. Scattered dots represent the whole dataset (with jitter). Lines and gold diamonds are the same as in Figure 4A.
